# Isolated splenic involvement in hydatid disease: A case report

**DOI:** 10.1016/j.radcr.2025.01.033

**Published:** 2025-01-23

**Authors:** Sakshi Dudhe, Devyansh Nimodia, Gaurav V. Mishra, Pratapsingh Hanuman Parihar, Paritosh Bhangale, Anjali Kumari, Rishitha Kotla

**Affiliations:** a Department of Radiodiagnosis, Datta Meghe Institute of Medical Sciences, Sawangi, Wardha, Maharashtra, India 442001; b Department of Psychiatry, Datta Meghe Institute of Medical Sciences, Sawangi, Wardha, Maharashtra, India 442001

**Keywords:** Spleen, Hydatid, Splenic hydatid, CT scan, Rare, Hydatidosis

## Abstract

Isolated involvement of spleen in hydatid disease is relatively uncommon occurrence. The spleen ranks as the third most frequently affected organ in this condition, liver primarily being the most commonly affected. When the disease manifests in the spleen, it typically presents with nonspecific symptoms, complicating the diagnostic process for healthcare professionals. Agricultural regions have endemicity for hydatid disease and the parasitic infection is caused by Echinococcus granulosus, specifically when occurs in splenic parenchyma, it is referred to as splenic hydatid disease (SHD). SHD has an incidence rate reported between 0.5% and 6.0% within the context of abdominal hydatidosis. Surgical intervention constitutes the primary modality of treatment, whereas the laparoscopic technique, when applicable, is deemed secure and presents the benefits associated with laparoscopic procedures. The conventional intervention entails either total or partial splenectomy performed via an open surgical approach. We present a case of 60-year-old female patient who was diagnosed with splenic hydatid cyst using Computed tomography (CT) scan.

## Introduction

Splenic hydatid cyst (SHC) is exceedingly rare form of hydatid contributing approximately 4% of cases of abdominal hydatid disease [1]. Berlot first described splenic hydatidosis in 1790 during an autopsy. 50%-80% of splenic cysts show echinococcal origin, especially in endemic areas [2]. By accidental ingestion of contaminated food and water which has eggs of the tapeworm, human get infected. Generally, SHC are asymptomatic or present with nonspecific symptoms. Patients with SHC can be asymptomatic for as long as 5 up to 20 years before the diagnosis is made [3].

The larval form of Echinococcus granulosus, commonly referred to as the dog tapeworm, instigates the condition known as Hydatid Disease (HD). Following the ingestion of hydatid eggs, multiple layered cyst is formed, that evolve into a hydatid cyst, which exhibits a growth rate ranging from 0.3cm to 1cm per annum. The initial clinical manifestation indicative of splenic hydatid disease typically involves an inadvertently discovered mass within the abdominal region, predominantly located in the left hypochondrium, with a lesser frequency observed in the epigastrium.

Pain, generally characterized as a dull aching pain, often represents the first clinical symptom. Symptoms such as dyspepsia, constipation, dyspnea, due to elevation of the left diaphragm, may also be present. The rupture of splenic hydatid cysts, along with subsequent complications such as anaphylaxis and calcification of the cyst, may arise. Given the potential for spontaneous or traumatic rupture, surgical intervention is the preferred treatment approach for splenic hydatid cysts. Preserving surgical techniques like partial splenectomy, present a risk for recurrence and postoperative hemorrhage; conversely, total splenectomy increases susceptibility to sepsis, particularly in pediatric patients, and thus should be approached with caution [4].

## Case presentation

**Fig. 1:**
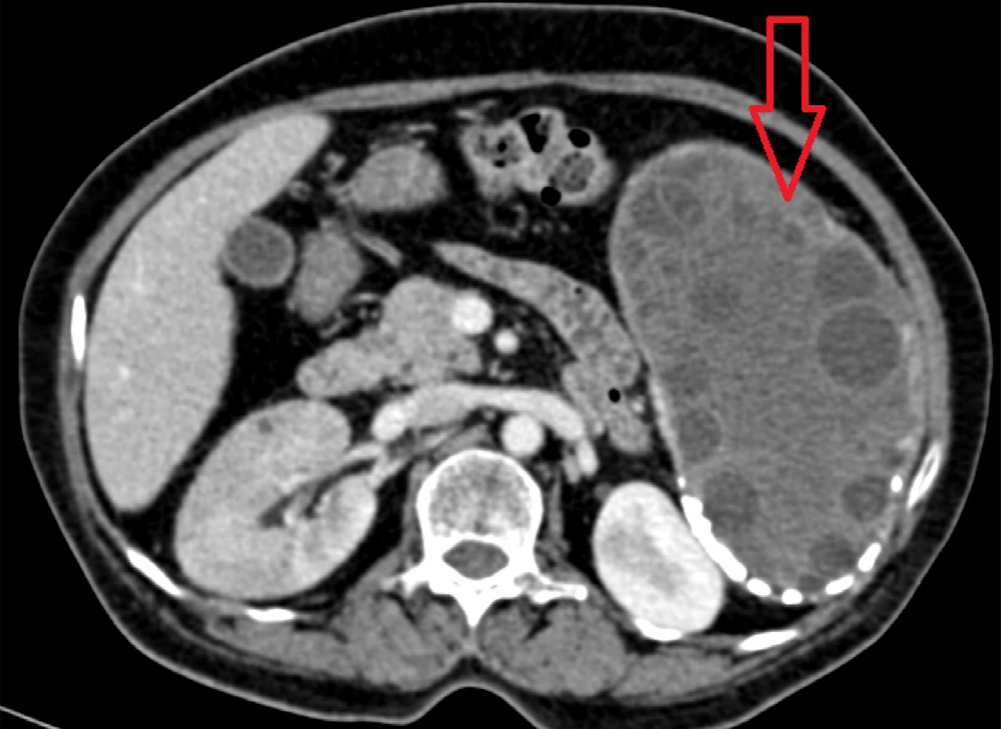
CT scan axial window showing multiloculated hypodense cyst multiple peripherally located daughter cysts. Wall of the lesion was partially calcified. The lesion measured 14 × 7.7 × 13.2cm.

**Fig. 2:**
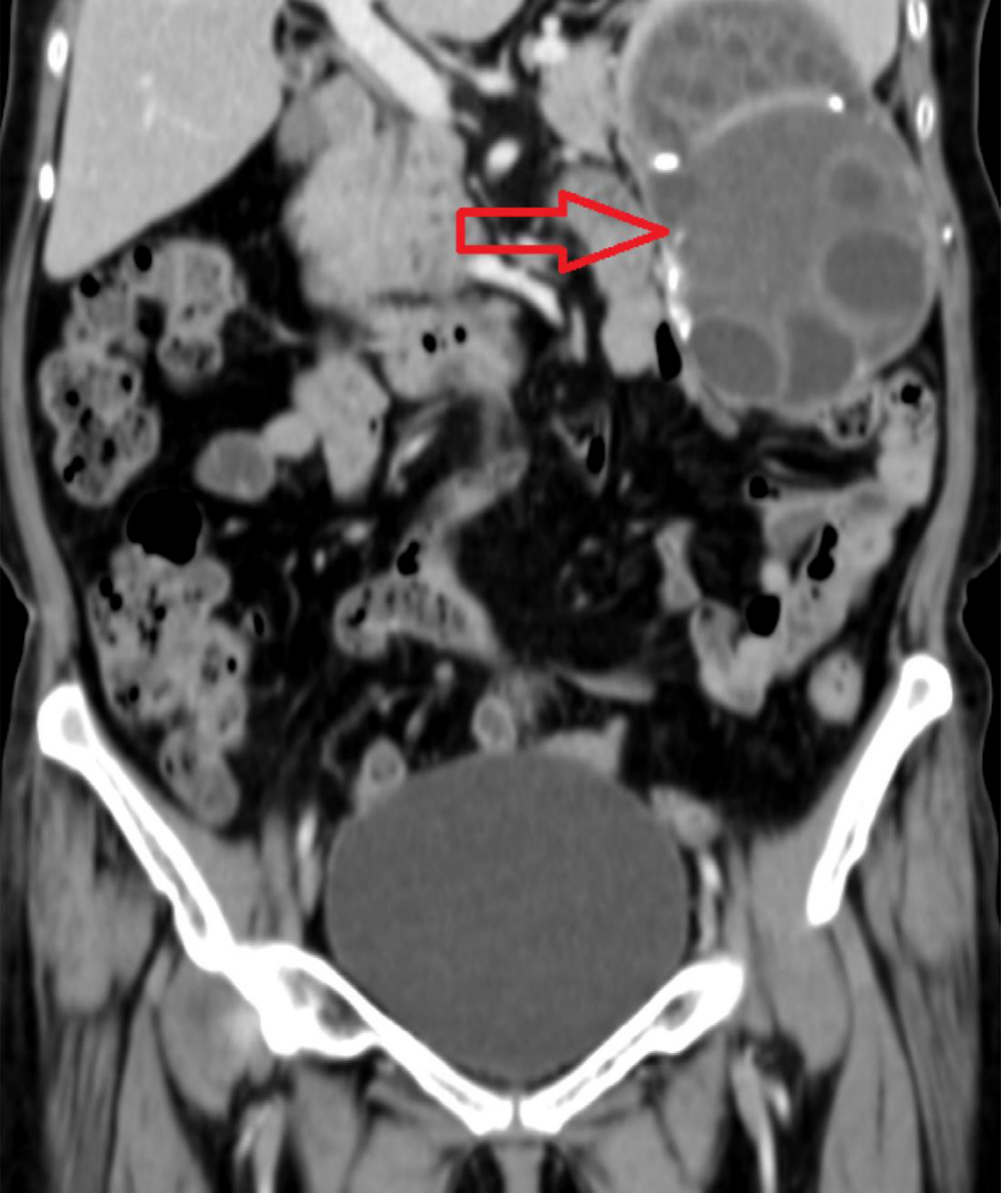
CT scan coronal window showing multiloculated hypodense cyst multiple peripherally located daughter cysts. Wall of the lesion was partially calcified. The lesion measured 14 × 7.7 × 13.2cm.

**Fig. 3:**
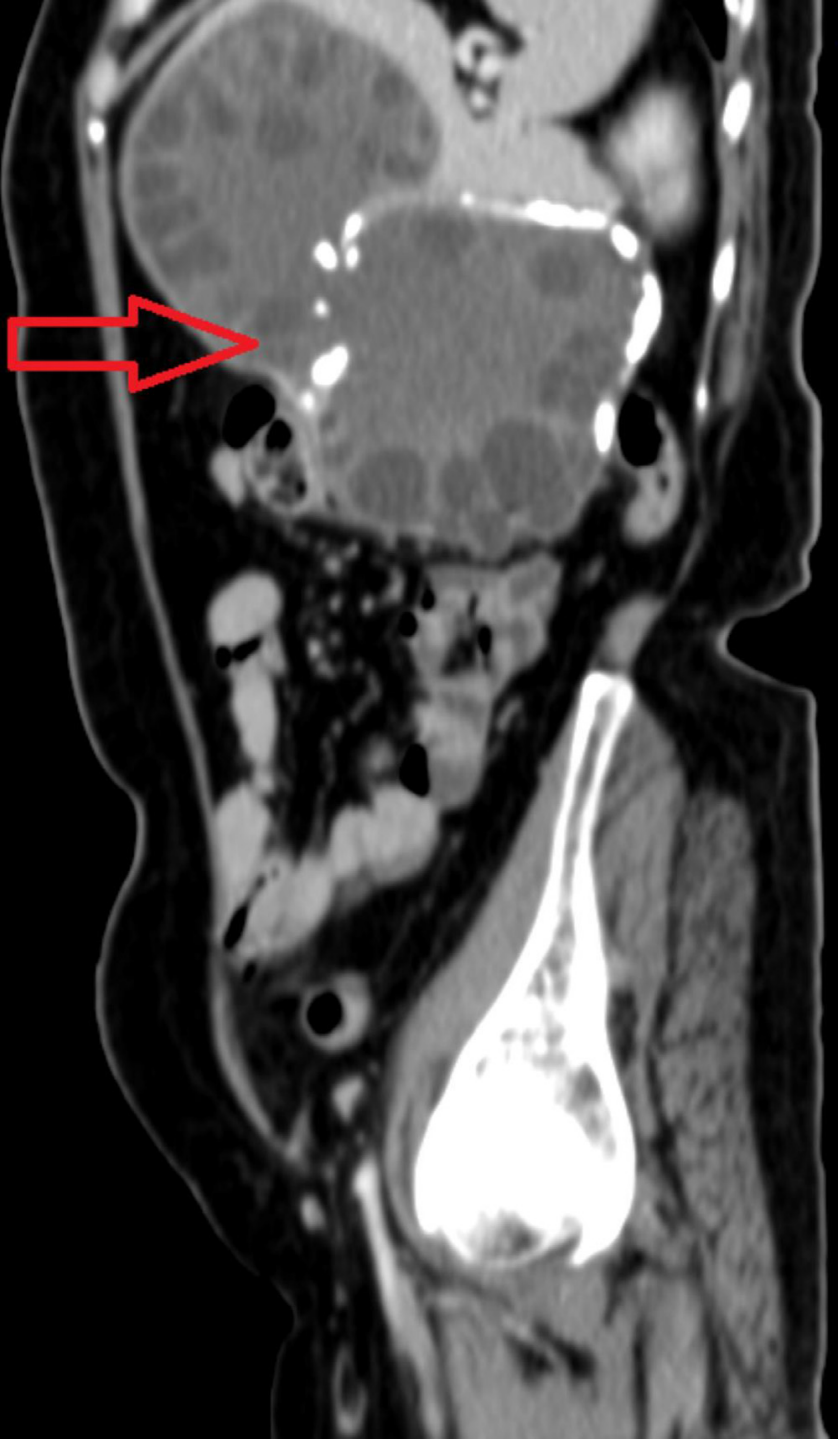
CT scan sagittal window showing multiloculated hypodense cyst multiple peripherally located daughter cysts. Wall of the lesion was partially calcified. The lesion measured 14 × 7.7 × 13.2cm.

A 60-year-old female presented to surgery outpatient department with pain in abdomen which was more on the left side and specifically in left hypochondrium. The nature of the pain was dull and dragging in nature. The patient also complained of feeling feverish for one month. Routine laboratory studies were normal. Serologic tests for E. granulosus by immunoblot assay were positive. An abdominal ultrasound revealed enlarged spleen measuring 18cm and a hypoechoic cystic lesion with internal tiny cysts within. Hydatid disease was first differential diagnosis, correlating with the history of pet dog at home. Imaging investigation with abdominal CT scan was done. It showed giant multiloculated hypodense cyst with internal solid component and multiple peripherally located daughter cysts as shown in Figs. 1, Fig. 2, Fig. 3. Wall of the lesion was partially calcified. The lesion measured 14 × 7.7 × 13.2cm. The lesion was classified as CE 3B by WHO classification.

Patient was started on albendazole 400mg oral tablet. It was given for 3 weeks before surgery. Indirect hemagglutination (HAI) was done, which was positive for Echinococcus species. Open splenectomy was performed. Histopathological analysis of the specimen proved it to be Hydatid cyst.

The procedure was well tolerated by the patient. She was discharged on the 5th postoperative day. Albendazole therapy with dose of 10mg/kg/day was given for 3 months postsurgery. A year later the patient was symptom free and had no long-term complications during the follow-up.

## Discussion

Abdominal hydatidosis is a type of cystic echinococcal zoonoses. Goeze was the first one to report it in 1782. It is usually known to affect sheep and cattle. Humans act as intermediate hosts within the developmental cycle of echinococcal parasite. This case is especially notable for documentation since splenic hydatid cysts are very uncommon, provide diagnostic problems, and develop seldom within the spleen [5]. Few hypotheses have been implicated in the etiopathogenesis of this disease. It includes an arterial route, a portal vein route by retrograde extension, rupture of hydatid cysts in other abdominal organs and systemic dissemination of parasite. Nonparasitic cysts in spleen constitute merely 10% and are rare [6].

Splenic hydatid cysts generally shows no significant symptoms. When the cyst grows bigger in size, the patient may present with a pain and discomfort in the left hypochondrium region. Other presentations include compression of renal artery, hypertension or cyst rupture into the adjacent organs. Imagining findings includes cyst wall calcification, daughter cysts and detached membrane [1]. Complications include secondary infection and fistulization to surroudning organs, supra-diaphragmatic region. Only 2% of all patients with echinococcosis have splenic hydatidosis [7].

Plain radiograph, Ultrasound, Computed Tomography, and Magnetic Resonance Imaging are used for diagnosis of the Splenic hydatidosis. Radiographs may reveal egg-shell-type calcification in the spleen. Ultrasound may reveal simple cysts of variable size with thickened wall. Daughter cysts may appear as hypoechoic on ultrasound, hypodense on CT, and low to intermediate signal intensity on T1- and high signal intensity on T2-weighted MRI. 90%-95% sensitivity of ultrasound is noted in detecting daughter cysts, hydatid membranes or sand. Daughter cysts show internal septations. The diagnosis of hydatid disease is predominantly established through noting folded inner cyst wall, the detachment of the hydatid membrane from the cyst wall, or the presence of hydatid sand. On ultrasonography and computed tomography, the observation of undulating membranes, which are pathognomonic, manifests as serpent signs. A subsequent collapse of the cyst may present as a whirl sign. Water-Lily sign denotes a collapse of the endocyst layer, whereby the inner cyst lining descends into the fluid residing in the dependent portion of the cystic lesion, thereby producing an appearance reminiscent of debris suspended on a fluid layer within the cyst. The differential diagnosis encompasses epidermoid cysts, pseudocysts, substantial solitary abscesses or hematomas, intrasplenic pancreatic pseudocysts, and cystic neoplasms of the spleen [2]. Hydatid cyst fluid lipoprotein antigen B (AgB) derived from E. granulosus is recognized as the most specific native or recombinant antigen for the purpose of immunodiagnosis [8].

Open or laparoscopic surgery is the most commonly used treatment of choice. For inactive superficial cysts at polar region of the spleen, conservative surgical techniques are used. They include partial splenectomy, enucleation of cyst, deroofing with omentoplasty, internal drainage with cystojejunalanastomosis, and external drainage can be done for preserving splenic parenchyma. For larger cysts in adults, splenectomy is done. younger patients. Limited splenic excision may be ideal for younger pediatric patients with increased pneumococcal titres to prevent postsplenectomy sepsis due to nonvaccination. Intraoperative scolicidal agents like cetrimide, 0.5% silver nitrate, hypertonic saline and alcohol for killing daughter cysts may be used. Post-therapy, recurrence screening and postoperative follow-up are necessary [9].

The principal objective of the surgical intervention is to prevent complications, eradicate the localized pathology. The incidence of mortality, morbidity, and postoperative recurrences can be reduced surgically. Both total splenectomy and spleen-preserving surgical techniques can reach these objectives. In the cases of total splenectomy, the parasitic components are entirely removed, thus eliminating associated complications. Adjunctive antiparasitic therapy further reduces the risk of recurrence [10].

## Conclusion

Rarely encountered splenic hydatid cysts represent a significant clinical challenge due to their potential complications and the need for tailored management strategies. Timely diagnosis through imaging and serological tests, combined with a multidisciplinary approach, is important for optimal outcomes. Surgical treatment remains the definitive treatment for most cases, especially for large or symptomatic cysts, with a preference for spleen-preserving techniques whenever possible. This case underscores the importance of considering hydatid disease in the differential diagnosis of splenic cystic lesions, particularly in endemic regions.

## Patient consent

Informed and written consent was obtained from the patient.

## Authors' contribution

SD and DN was involved in providing clinical details of the patient. PHP discussion on the pathology. GVM accumulated the results of the patient's radiological investigations. PB, AK and RK was involved in collecting images and formatting data. All authors have read and approved the manuscript.

## Ethics approval and consent to participate

written consent taken.

## Availability of data and material

N/A.
